# Numerical and Experimental Study of Gas Phase Nanoparticle Synthesis Using NanoDOME

**DOI:** 10.3390/nano13081317

**Published:** 2023-04-08

**Authors:** Giorgio La Civita, Edoardo Ugolini, Nicola Patelli, Alberto Piccioni, Andrea Migliori, Luca Pasquini, Emanuele Ghedini

**Affiliations:** 1 Department of Industrial Engineering, University of Bologna, v. del Risorgimento 2, 40136 Bologna, Italy; edoardo.ugolini3@unibo.it (E.U.); emanuele.ghedini@unibo.it (E.G.); 2 Department of Physics and Astronomy, University of Bologna, v. Berti-Pichat 6/2, 40127 Bologna, Italy; nicola.patelli@unibo.it (N.P.); alberto.piccioni@unibo.it (A.P.); luca.pasquini@unibo.it (L.P.); 3 Institute for Microelectronics and Microsystems, National Research Council, via Gobetti 101, 40129 Bologna, Italy; migliori@bo.imm.cnr.it

**Keywords:** gas phase synthesis, inert gas condensation, NanoDOME, SimDOME, CFD, nanoparticle, PBM, MoM, classical nucleation theory, multiphysics

## Abstract

Nowadays, with the rocketing of computational power, advanced numerical tools, and parallel computing, multi-scale simulations are becoming applied more and more to complex multi-physics industrial processes. One of the several challenging processes to be numerically modelled is gas phase nanoparticle synthesis. In an applied industrial scenario, the possibility to correctly estimate the geometric properties of the mesoscopic entities population (e.g., their size distribution) and to more precisely control the results is a crucial step to improve the quality and efficiency of the production. The “NanoDOME” project (2015–2018) aims to be an efficient and functional computational service to be applied in such processes. NanoDOME has also been refactored and upscaled during the H2020 Project “SimDOME”. To prove its reliability, we present here an integrated study between experimental data and NanoDOME’s predictions. The main goal is to finely investigate the effect of a reactor’s thermodynamic conditions on the thermophysical history of mesoscopic entities along the computational domain. To achieve this goal, the production of silver nanoparticles has been assessed for five cases with different experimental operative conditions of the reactor. The time evolution and final size distribution of nanoparticles have been simulated with NanoDOME by exploiting the method of moments and population balance model. The validation is performed by comparing NanoDOME’s calculations with the experimental data.

## 1. Introduction

Bottom-up methods for NPs synthesis can be divided into two broad categories based on the phase in which the nucleation and growth of NPs occurs, namely the liquid phase and gas phase. In gas phase nanoparticle synthesis (GPNS), atomic or molecular vapours are brought into a condition of supersaturation that leads to the nucleation of NPs. The production of vapours is typically started from solid precursors via thermal evaporation, laser ablation, or plasma processing. A recent comprehensive review of gas phase production of nanomaterials can be found in [1]. The process modelled in the present paper exploits the thermal evaporation of metals within a chamber filled with pure helium (He), which cools down the metal vapors, leading to their supersaturation. This approach belongs to the class of physical GPNS in the sense that no chemical reactions intervene to modify the NPs composition with respect to the precursor. The method, also known as inert gas condensation (IGC), was described in the 1970s by Granqvist and Buhrman for several pure metal NPs [2] and was later pioneered by Gleiter for the preparation of bulk nanocrystalline solids via compaction of NPs under high vacuum [3]. Several authors committed their efforts to the experimental investigation of the thermophysical evolution of NPs [4]. It is well known that the three main options to increase the average NPs diameter are as follows: (i) increase the evaporation temperature; (ii) increase the pressure or the atomic mass of the inert gas; (iii) decrease the mass flow rate of the inert gas. However, a more quantitative knowledge of the relation between thermodynamic parameters and NP size distribution, as well as a predictive capability adapted to specific experimental arrangements, are required, and this is where accurate numerical modelling becomes of the utmost importance. Numerical modelling-wise, several articles have been published over the years. Various sets of stochastic and deterministic models for multi-physics assessments of NP size distribution and for tracking the thermophysical evolution of NPs have been proposed. The current state-of-the-art research for GPNS suggests the population balance (PBM) [5] and moments methods (MoM) [6] as efficient and reliable tools for GPNS modelling. Moreover, recently, multi-physics linked models have emerged as powerful tools to intimately investigate GPNS. The integration of finely tuned multi-scale numerical models with experimental data has gained increasing importance and expanded its field of applications. For instance, Hongyu L. et al. [5] performed a study similar to this work, in which they proposed a reactive kinetics solver coupled with PBM and computational fluid dynamics (CFD); they analysed and validated the model with the support of experimental data from a micro-tubular reactor for the chemical production of silver NPs. The quadrature method of moments and kinetic theory of granular flow was used to solve the model and obtain the final particle size distribution (PSD).

In this work, numerical and experimental approaches are integrated for both validation and methodology. The adopted experimental reactor’s fluid dynamics are linked to a mesoscopic model. The goal is to provide a more detailed picture of NP thermophysical evolution from both the qualitative and quantitative point of view during IGC processes. Moreover, the proposal of the NanoDOME framework as a reliable modelling tool represents an important goal of this research. In order to achieve our stated goals and for the particular experimental setup adopted for this work, the NPs are transported both by a forced He flow and by natural convection toward a collection cylinder, which is internally cooled by liquid nitrogen. The He pressure lies in the hundreds of Pa range, while the He flow spans the range 10–120 scc/min. Even though the influence of these parameters on the average NP size is known from a qualitative point of view, a deeper understanding of the formation and growth mechanism of NPs together with the predictive capability offered by reliable modelling tools are highly desirable to guide the development of controlled nanomaterial architectures.

The current paper aims to propose the NanoDOME software, developed under the H2020 European Project NanoDOME G.A. n. 646121, as a functional and consistent engineering toolkit for GPNS modelling in a low pressure and high temperature gradient environment. Furthermore, NanoDOME can be a valid tool to simulate the nanoparticle formation process using gas phase synthesis and assess the NPs’ most relevant properties, thus, shortening the development process of novel gas phase production routes and nanomaterials.

Another main purpose of this work is to validate and finely tune the model, characterizing the crucial thermodynamic condition for particles’ mean diameter distribution during GPNS. Furthermore, validation is performed by comparing numerical results with experimental data of silver NPs, obtained from the analysis of NPs using electron microscopy.

## 2. Materials and Methods

Operative conditions of the experimental benchmark cases and their partitioning into three different subclasses will be outlined in this section, describing the design of experiment (DOE). A multi-scale approach is also investigated alongside the linking process between NanoDOME and OpenFOAM© [7]. Moreover, mesoscopic and continuum scales are discussed. Lastly, a general overview on the physical mathematical formulation of the whole phenomenon is reported. The model includes the general aerosol dynamic equation (GDE), solved using the MoM, as performed by Xiong et al. in [8] and Galleni et al. in [6], together with the stochastic PBM. For the sake of clarity, it is crucial to remember that the multi-scale simulations performed in this work do not require a chemical solver due to the nature of the components, also available in NanoDOME. In fact, simulations are carried out for a low pressure and high temperature gradient reactor, with He as the carrier gas. Hence, due to the lack of reactive species, chemical events, such as oxidation, can be neglected. Lastly, the fluid dynamics of the continuum scale account for the buoyancy effects (e.g., natural convection flows at a large Rayleigh number), under the Oberbeck–Boussinesq assumption. For any further references and deepening of each methodologic choice of the model, please refer to [9].

The NanoDOME and OpenFOAM© software are publicly available with an L-GPL v3.0 license and can be downloaded at: https://github.com/nanodome/nanodome-public (accessed on 7 March 2023) and https://www.openfoam.com/news/main-news/openfoam-v2212 (accessed on 7 March 2023), respectively.

### 2.1. Mesocopic Model Terminology

In the current section, the terminology that describes the complete set of mesoscopic entities involved in the gas phase synthesis process and used for the NanoDOME mesoscopic model definition is reported. Such terminology is used within the scope of the present study and refers to the terminology used by Strappaveccia et al. in [10]. Further documentation on the model terminology developed during the NanoDOME and adopted in the SimDOME projects can be found in [9]. In this study, the term nanoparticle will be used to refer to objects that span from a single particle to complex agglomerates formed by several aggregated primary particles [11]. Following the definitions of Wang et al. [12] and of Bandyopadhyaya et al. in [13], a NP is considered as a solid particle, the size of which is below 200 nm. Furthermore, NPs are commonly subdivided into three categories:

- Primary particles: the tiniest identifiable single particle. They often present a single crystalline structure. Their size is between 5 nm and 50 nm.
- Agglomerates: more complex ensemble of primary particles held together by weak bonds (e.g., Van der Waals force). For such structures, the total surface area coincides with the sum of single particle areas. Thermodynamically, it is fundamental to underline that agglomeration is a reversible process.
- Aggregates: assemblies of partially sintered primary particles, held together by strong bonds (covalent, metallic, ionic). The total surface area of such structures is smaller than the total surface area of the original primary particles.

### 2.2. Linking Multi Scaled Approach

The proposed framework can capture the multi-scale physics involved in the phenomenon, e.g., mesoscopic and continuum scale. To achieve this, computational fluid dynamics (CFD) data from the reactor model, in the form of streamlines of the gas phase (GP) thermodynamic properties, are linked to the mesoscopic simulation (NanoDOME). Considering the great span between the timescales involved, this choice provides lower time demands and complexity than a direct coupling approach.

The linking process between the mesoscopic and continuum scales proposed in NanoDOME is based on parcels. Parcels are massless numerical Lagrangian particles under the influence of the flow field. Every parcel can follow the evolution of the thermophysical properties. CFD simulations are performed by the open-source toolbox OpenFOAM© [7]. An implemented extension for gas phase fluid dynamic linking to NanoDOME has been developed. The extension drives the linking process in a way that can be summarized as follows:

I. Thermodynamic condition (boundary and initial) initialization on each instance for CFD steady simulations. Steady simulations are performed, storing data in the form of streamlines.
II. Every streamline is passed to a different NanoDOME instance, which performs the simulation.
III. An average value of the obtained solutions is computed as the final prediction.

**Figure 1:**
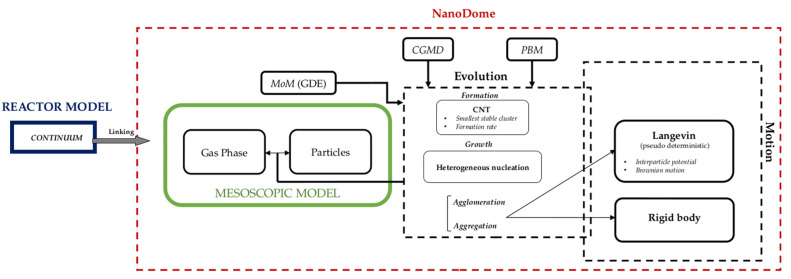
Conceptual workflow diagram of the linking and mesoscopic system modelling adopted in this work.

Figure 1 shows a comprehensive workflow diagram which provides an overview of the linking process between the mesoscopic and continuum scale and the exploited submodules for the evolution of particles and their motion, as described in the next section.

The reactor’s fluid dynamics lead to a set of nine streamlines for each of the experimental conditions summarized in Table 1. Streamlines are evaluated in a steady-state regime, while the evolution of the thermophysical properties given by nano-synthesis phenomena is performed in a transient regime. For the MoM, the final prediction on aggregates and particle dimension distribution is considered as an average value among each streamline. On the other hand, the PBM method, starting with an initial value of the PSD, stochastically collides particles, tracking the evolution of their geometrical characteristics.

### 2.3. Mesoscopic System

The mesoscopic system is a set of particles in free molecules’ gaseous phase, namely the gas phase (GP). The model can predict both primary particles and agglomerates. The following are the physical assumptions which the model is based on:

- NPs range in size from 1 nm to 200 nm.
- Computational domain size length $l_{v},\left(1<l_{v}<10\right)\;\mu m$.
- $\left(10^{2}<N_{p}<10^{5}\right)$ number of NPs considered.
- Time scaling of $\left(1<t_{s}<100\right)\;\mathrm{ms}$.
- The fundamental object of the mesoscopic scale model is the minimum thermodynamically stable structure, previously defined as a primary particle.
- A primary particle has a spherical shape.

**Figure 2:**
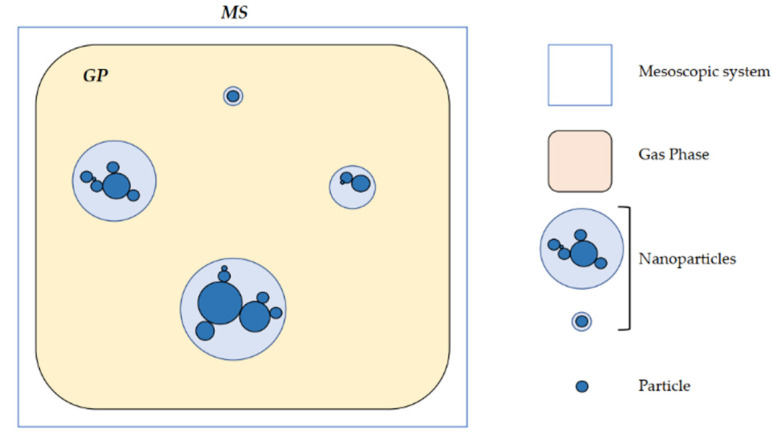
Mesoscopic system schematization.

In this work, the mesoscopic system is defined as a population of particles $p_{i}$ $i\in \left\{1,\ldots ,N\right\}$ where N is the total number of particles in the mesoscopic system inside a gas phase composed by free molecules, as represented in Figure 2. A nanoparticle ${\mathrm{NP}}_{i}$ can bind to other particles by weak (e.g., interparticle potential) or strong interactions (e.g., hard bonds or sintering). The former characterizes agglomeration, while the latter characterizes aggregation. The primary particle consists of a single particle formed because of nucleation. Therefore, the validity of the ${\mathrm{NP}}_{i}\cap {\mathrm{NP}}_{j}=\emptyset$ relation holds for two different NPs, namely ${\mathrm{NP}}_{i},{\mathrm{NP}}_{j}$. An aggregate structure is a subpart of n particles belonging to a nanoparticle NP, $\left\{p_{j}\right\}\subset \mathrm{NP}$ connected by partial sintering.

The gas phase, as stated above, can be defined as free molecules and atoms, characterized by a set of thermodynamic quantities (e.g., temperature, pressure, molar concentration, etc.). Furthermore, free molecules and atoms lie below the mesoscopic model characteristic length. The total number of molecules is $N_{\mathrm{gas}}$, and the molar concentrations are $C_{s}\left(t\right)$ with $s\in \left(1,\ldots ,S\right)$, where S is the total number of species in the system.

#### 2.3.1. Particles Formation and Motion

The processes which lead to the formation and growth of the NPs are, respectively, modelled with the classic nucleation theory (CNT) and heterogeneous nucleation. It is important to underline that for MoM the particles’ evolution is already considered by the GDE. For the PBM method, the number of molecules for the smallest stable cluster, defined in Equation (1) according to the classic nucleation theory, gives the thermodynamic threshold to give the former event under thermal equilibrium conditions (e.g., no radiative exchange). Equation (1) is as follows:

(1) $$j_{s}={\left(\frac{8{\pi r}_{0,s}^{2}\sigma_{s}}{3k_{B}\mathrm{Tln}\left(S_{s}^{\mathrm{sat}}\right)}\right)}^{3}$$

where $r_{0,s}$ is the radius of the s molecule, $\sigma_{s}$ is the superficial tension of the liquid, $k_{B}$ is the Boltzmann constant, T is the gaseous phase temperature, and $S_{s}^{\mathrm{sat}}=p_{s}/p_{s}^{\mathrm{sat}}$ is the supersaturation ratio. The formation rate $R_{s}$ of primary particles, based on [14] and [15], is given by Equation (2):

(2) $$R_{s}=n_{s}n_{s}^{\mathrm{sat}}v_{s}\sqrt{\frac{2\sigma }{m_{s}\pi }}e^{\left(\Theta -\frac{4\Theta^{3}}{27\left(\ln{\left(S_{s}^{\mathrm{sat}}\right)}^{2}\right)}\right)V_{\mathrm{crit}}}$$

where $n_{s}$ is the species s concentration in the gas phase, $n_{s}^{\mathrm{sat}}$ is the concentration at the saturation state, $v_{s}$ is the volume of species s, $V_{\mathrm{crit}}$ is the critical gaseous medium volume, $m_{s}$ is the mass, and Θ is the normalized surface tension defined as $\theta =4{\pi r}_{0}^{2}\sigma_{S}/k_{B}$. Particle motion is described by the Langevin equation of motion. Aggregates are considered as rigid bodies with a centre of mass $x_{\mathrm{MC}}$, mass $m\left(\mathrm{AG}\right)$ under the influence of forces due to interparticle potential and Brownian behaviour. The Brownian force is evaluated as the sum of the same forces applied on each particle of the aggregate.

#### 2.3.2. GDE and MoM Method

The GPNS process can be described with the equation proposed by Xiong et al. in [8], the generalized aerosol dynamic equation GDE in Equation (3). The model, accounting for collision-driven coalescence, assumes a lognormal distribution of particles population, as follows:

(3) $$\frac{\partial n\left(v_{p}\right)}{\partial t}+u\frac{\partial \left(\mathrm{Gn}\right)}{\partial v_{p}}-I\left(v_{p}^{c}\right)\delta \left(v_{p}-v_{p}^{c}\right)=\frac{1}{2}\int_{0}^{v_{p}}\beta \left(v_{p}-v_{p}^{\prime },v_{p}^{\prime }\right)n\left(v_{p}-v_{p}^{\prime }\right)n\left(v_{p}^{\prime }\right){\mathrm{dv}}_{p}^{\prime }-\int_{0}^{\infty }\beta \left(v_{p},v_{p}^{\prime }\right)n\left(v_{p}^{\prime }\right){\mathrm{dv}}_{p}^{\prime }$$

where n is the PSD, u is the gaseous mixture’s velocity, $v_{p}$ is the particle volume, D is the diffusion coefficient of the particle, I is the nucleation rate, δ is the Dirac distribution function, G is the heterogeneous condensation growth rate, and β is the interpolative collisional frequency function. The c superscript refers to the critical state. The first term of the left-hand side (LHS) indicates the rate of change in particle concentration in the volume interval v+Δv. The second term of the LHS accounts for the loss or gain of particles by condensation, with rate G. The last term of the LHS describes the formation of new particles at their critical volume $v_{p}^{c}$, with nucleation rate I. Terms belonging to the right-hand size consider gain and loss of particles with the volume incremental range v+Δv by coagulation.

In the following paragraph, the MoM method is briefly described as a solving tool for GDE, provided by NanoDOME for predicting NPs’ average dimension distribution. The MoM method requires a slightly different formulation of the GDE, as in Equation (4), based on the definition of three moments of the PSD as in Equation (5):

(4) $$\frac{\partial M_{k}}{\partial t}={\left[\dot{M_{k}}\right]}_{n}+{\left[\dot{M_{k}}\right]}_{c,e}+{\left[\dot{M_{k}}\right]}_{c}+{\left[\dot{M_{k}}\right]}_{d}$$

(5) $$M_{k}=\int_{0}^{\infty }v_{p}^{k}n\left(v_{p}\right){\mathrm{dv}}_{p}$$

where $k=\left(0,1,2\right)$, $v_{p}$ stands for the particle volume, and $n\left(v_{p}\right)$ is the PSD. To achieve closure, the method assumes a lognormal distribution for the nanoparticle size. The moment $M_{0}$ describes the overall nanoparticles’ concentration, the first moment $M_{1}$ represents their total volume, and $M_{2}$ describes the light scattered by nanostructures. The terms $\left[\dot{M_{k}}\right]$ indicate the production rates, respectively, due to nucleation, condensation and evaporation, coagulation, and diffusion mechanisms. The system mathematical closure is assessed with the conventional definition of geometric standard deviation, Equation (6), and geometric mean volume, Equation (7), and their dependence on the moments:

(6) $$\ln\left(\sigma_{g}\right)=\frac{1}{9}\ln\left(\frac{M_{0}M_{2}}{M_{1}}\right)$$

(7) $$v_{g}=\frac{M_{1}^{2}}{M_{0}^{\frac{3}{2}}M_{2}^{1}}$$

#### 2.3.3. Stochastic Approach—PBM Method

The PBM method performs trivially as a balance on the number of particles that share well defined properties, e.g., size. Since the method needs an initial PSD, in this work we assumed a lognormal distribution of the initial population, as was previously performed by Xiong et al. in [8]. As stated, the necessity to evaluate a fractal characteristic dimension arises. Such a quantity can be defined as follows, as previously carried out in [9]:

(8) $$D_{\mathrm{AG}}=\frac{\ln\left(N_{r}\right)}{\ln\left(d_{c}\right)/d_{p,\mathrm{av}}},\;N_{r}=\frac{S^{3}}{36{\pi v}^{2}}$$

(9) $$\left\{\begin{array}{c} d_{c}=2\sqrt{\frac{5}{3}}R_{G}\\ d_{p,\mathrm{av}}=\frac{6v}{S} \end{array}\right.$$

where $D_{\mathrm{AG}}$ is the fractal dimension, $N_{r}$ is the reduced number of particles of the aggregate, $d_{c}$ is the collision diameter, $d_{p,\mathrm{av}}$ is the average diameter of the particles, S is the total area, and v is the total volume of the aggregate, considered as summatory on all particles. As affirmed previously, this method is based on stochastic driven collisions with pre-defined fractal dimensions.

### 2.4. Reactor Model—Continuum Scale

The fluid dynamic involved in this phenomenon can be described with the Navier–Stokes transport equation for a turbulent and incompressible flow. The flow is modelled with the following assumptions:

- Steady-state regime.
- Absence of thermal radiative exchange.
- Accounting for density-driven buoyancy effects.
- Turbulent flow regime.
- Pressure and temperature related thermophysical properties.

To describe the turbulent nature of such physics, the k-ω SST model by Menter et al. [16], natively implemented in OpenFOAM, is used. The steady-state simulations are carried out by the native OpenFOAM’s buoyantSimpleFOAM solver [5], used to compute the reaction chamber thermodynamic and fluid dynamic properties by means of a set of streamlines for each test case. Despite the gaseous nature of the GP and the convective nature of the flow inside the reactor, the gas flow is considered incompressible due to its subsonic regime. Pressure- and temperature-dependent thermophysical properties are retrieved from Janaf tables and the Sutherland transport model provided by OpenFOAM© [17].

**Figure 3:**
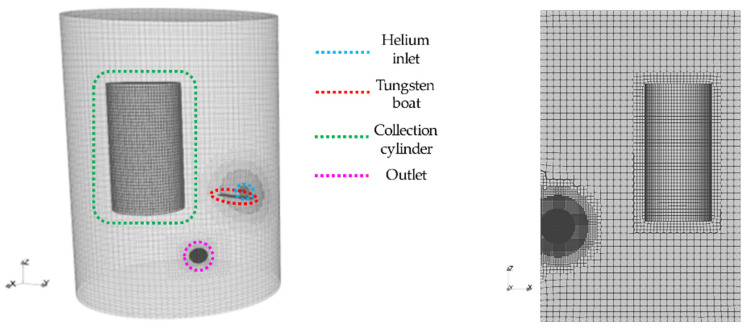
Simulated 3D geometry of the reactor, (**left**) frontal view and (**right**) xz-plane section.

The experimental apparatus sketched in Figure 3 consists in a cylindrical ultra-high-vacuum chamber made of stainless steel with an internal volume of 112 L. The evaporation of the metal precursor (silver granules, 0.7–1.5 mm, 99.99% pure, supplied by Balzers Materials) takes place within a Joule-heated tungsten boat connected to high current feedthroughs. The inlet of helium gas (99.9999% pure) is placed in the proximity of the tungsten boat, slightly above the melt. The helium flow, which is regulated and kept constant by a mass flow controller, is directed toward a hollow stainless steel cylinder that serves as the collection surface. The top of the cylinder is attached to a hollow feedthrough (not shown) that allows for filling the cylinder with liquid nitrogen from the outside as well as rotating it as needed. During operation, the pressure inside the chamber is kept constant by compensating the incoming helium flow with a mechanical rotary pump connected to the chamber via a metering valve. The apparatus has been used for the synthesis of several NP materials, such as Fe [18], Fe@FeOx core@shell [19], Mg-based composites [19], Fe-Co alloys [20], and TiO_2_ (through post-oxidation of Ti NPs) [21]. In this work, the synthesis of silver NPs by gas phase synthesis (i.e., inert gas condensation) under different temperature, pressure, and flow conditions was carried out with the aim of comparing experimental data with NanoDOME calculations. The silver vapour generated by the tungsten boat is carried by the helium gas flow and cooled as it approaches the cylindrical collection surface. When the vapour’s temperature becomes lower than the saturation temperature at the chamber’s pressure, NPs begin to form by condensation/nucleation and eventually sintering. One reason for this choice is that silver NPs are not prone to heavy oxidation upon air exposure; therefore, the NPs’ diameter determined ex situ after the synthesis is a faithful representation of the original size. Moreover, silver NPs display a peak in the optical absorption spectrum due to the localized surface plasmon resonance, the position of which correlates with the NPs’ size and shape. Therefore, the control of the NPs’ diameter via modelling-aided synthesis allows for tailoring the optical absorption spectrum of gas phase-condensed silver NPs.

The geometry has been firstly designed according to the experimental reactor topology inside a CAD software. Then, the resulting 3D model has been passed to the OpenFOAM’s natively available mesh tool “cfMesh”. The following measurements are the implemented mesh parameters: max cell size $CS_{max}=0.015\;m$, cold source refinement levels $RL_{cs}=1$, and refinement thickness $RT_{cs}=1\;\mathrm{mm}$, hot source and outlet gas refinement levels $RL_{og}=1\;\mathrm{mm}\;$, and refinement thickness $RT_{og}=0.0025\;m$. Local refinement has been applied to steep gradient local areas, such as the inlet carrier gas nozzle, and the hot and cold sources and the outlet have been refined. The choice of a highly detailed zone is justified by the need to capture the characteristic timescale of nucleation and sintering phenomena, which belong to the mesoscopic timescale.

### 2.5. Thermodynamic and Operative Conditions

Simulations of silver NPs’ formation are carried out for a specific set of thermodynamic operative conditions, the DOE of which is reported in Table 1. To characterize the effect of temperature, pressure, and carrier gas mass flow rate on the particles’ size, the DOE has been constructed by varying each parameter at a time. Hence, as the partitioning criterion, a set of three thermodynamic quantities controllable by the reactor has been chosen: hot source temperature $T_{h}$(K), the reactor pressure p(Pa), and helium mass flux $m_{He}$ (kg/s).

**Table 1:** DOE of the five operative conditions for the PBM and MoM, where p is pressure (Pa), $m_{He}$ is mass flow rate (kg/s), and $T_{h}$ is temperature (K). The Roman numerals between round brackets represent the subclass identifier for each of the three operative conditions (i: varying $T_{h}$; ii: varying $m_{He}$, and iii: varying p). The collection surface was cooled by liquid nitrogen in all cases.

Case	p (Pa)	$m_{He}\;(\mathrm{kg}/s)$	$T_{h}\;(K)$
1 (i)	260	1.8 $\times \;10^{-7}$	1260
2 (ii, i)	260	1.8 $\times \;10^{-7}$	1403
3 (iii, i)	260	1.8 $\times \;10^{-7}$	1466
4 (iii)	2600	1.8 $\times \;10^{-7}$	1466
5 (ii)	260	3.6 $\times \;10^{-7}$	1403

The expected effects of each parameter can be summarized as follows:

- Source temperature $T_{h}$ (K): By increasing the source temperature, the primary particle temperature is also increased, thus, boosting their tendency to form agglomerates because of sintering and coagulation. It must be pointed that the lower and upper limit are due to the solid precursor melting point and instrumental working limit.
- Inert gas mass flux $m_{He}$ (kg/s): The effect of the convective cooling load given by the helium flux strongly influences the thermophysical evolution of the mesoscopic entities. For a regular section nozzle, the relation $v\propto m_{He}/A_{noz}$ holds, where v is the gas velocity, $m_{He}$ is the mass flux, and $A_{noz}$ is the nozzle’s cross-section. Assuming constant gas density and cross-section of the nozzle lead to a linear dependence of the gas velocity on the mass flux, if $m_{He}$ increases, so does the convective cooling effect of helium.
- Reactor pressure p (Pa): The reactor operates at two different low-pressure conditions, typical for GPNS. For cases 2 ($p_{2}=260\;\mathrm{Pa}$) and 4 ($p_{4}=2600\;\mathrm{Pa}$), independently from the hot source temperature and carrier gas flux, higher pressure leads to an increase in the average number of collisions among particles inside the reactor. Hence, sintering and coalescence phenomena are more likely to occur. Therefore, an increase in the final distribution’s average diameter is expected.

### 2.6. Experimental Determination of NPs Size Distribution

**Figure 4:**
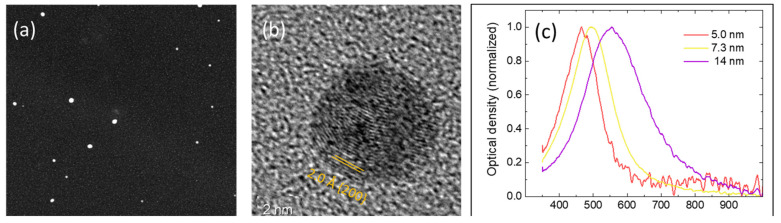
(**a**) Dark-field STEM image of silver NPs synthesized under the conditions denoted as case 1 (reference). (**b**) High-resolution image of a single silver NP showing the lattice planes belonging to the {200} family. (**c**) Optical absorption of silver NP ensembles with different average diameters as reported in the legend.

The NPs’ size distribution for the various cases were determined by transmission electron microscopy (TEM) and scanning electron microscopy (SEM). To this purpose, grids constituted by a holey carbon film on a copper mesh (for TEM), and ultra-smooth Si pieces (for SEM) were attached to the surface of the cylinder and exposed to the incoming NPs for about one minute. We used a Tecnai F20 TEM microscope operated at 200 kV, and a Zeiss LEO 1430 SEM. The results obtained with the two methods are consistent, although TEM is more accurate, especially for small NPs. Therefore, the experimental data and distributions reported in the following were determined from the analysis of several TEM images with the aid of the ImageJ software package. Figure 4a shows one exemplary TEM image of silver NPs, acquired in scanning TEM (STEM) mode using a high-angle annular dark field detector. Figure 4b displays a high-resolution TEM (HRTEM) image of a single silver particle; the lattice planes belonging to the {200} family with an interplanar spacing of 2.0 Å are clearly visible. Figure 4c illustrates the size-dependent optical absorption of silver NPs. For this measurement, the NPs were deposited on a glass substrate; the absorption peak redshifts with increasing average diameter, a well-known feature of the plasmon resonance.

## 3. Results

In this section, the results obtained from the linked OpenFOAM©–NanoDOME simulations are discussed. As reported previously in the methodology section, the continuum scale (experimental reactor model) evaluates a set of nine streamlines, tracking the evolution of several thermophysical properties of particles. For each test case of the DOE, the following quantities will be discussed in the next paragraphs:

- Number density (ND) weighted average diameter $\bar{d}$ over time in the computational domain (nm) and its standard deviation σ (STD).
- PSD for primary particles diameter (nm).

**Figure 5:**
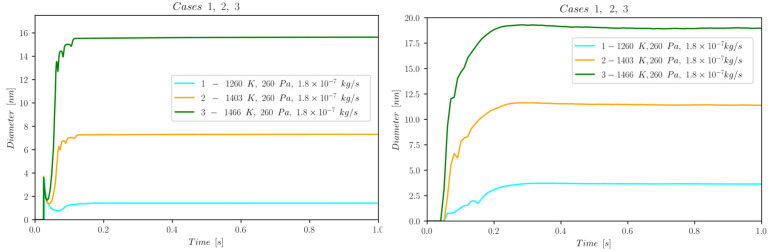
Temporal evolution of $\bar{d}$ for cases 1, 2, and 3 belonging to subclass i (temperature dependence); (**left**) MoM, (**right**) PBM.

**Figure 6:**
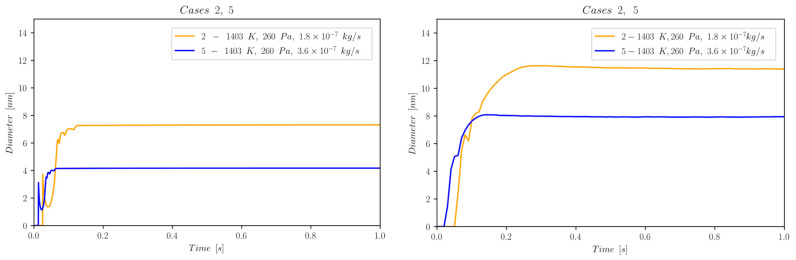
Temporal evolution of $\bar{d}$ for cases 2 and 5, belonging to subclass ii (helium flow-dependence); (**left**) MoM, (**right**) PBM.

**Figure 7:**
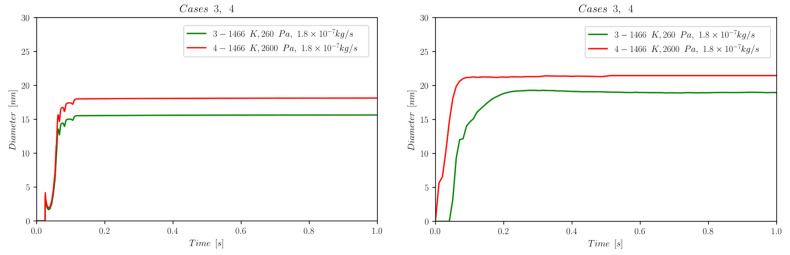
Temporal evolution of $\bar{d}$ for cases 3 and 4, belonging to subclass iii (pressure variation); (**left**) MoM, (**right**) PBM.

**Figure 8:**
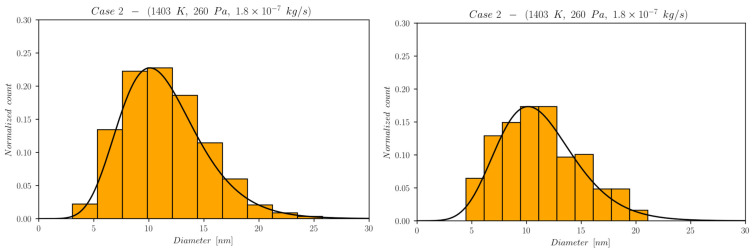
Final PSD for reference case 2; (**left**) PBM predictions and (**right**) experimental results.

**Figure 9:**
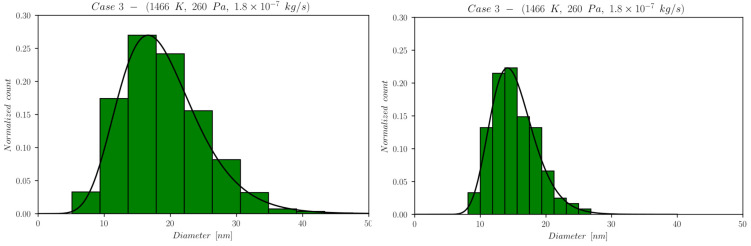
Final PSD for case 3; (**left**) PBM predictions and (**right**) experimental results.

**Figure 10:**
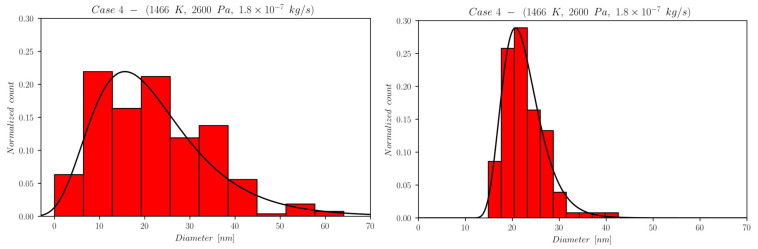
Final PSD for case 4; (**left**) PBM predictions and (**right**) experimental results.

**Figure 11:**
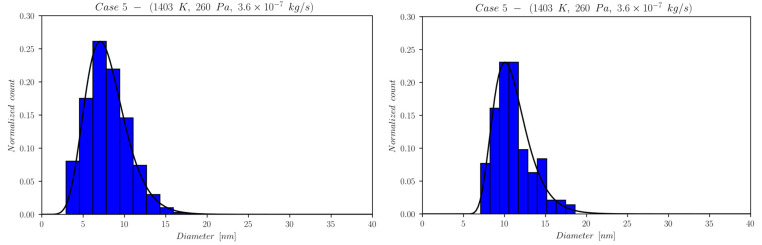
Final PSD for case 5; (**left**) PBM predictions and (**right**) experimental results.

The first set of data reports $\bar{d}$ as a function of time computed by NanoDOME. These time evolutions are shown in Figure 5, Figure 6 and Figure 7 for the three subclasses of the DOE, for both the MoM and PBM approach. The second set of data contains quantitative information on the final PSD obtained by PBM. The PSDs are displayed in Figure 8, Figure 9, Figure 10 and Figure 11 and compared to the experimental data using a normalized vertical scale histogram. The normalized particle counts ${\hat{n}}_{l}$ for a given bin of the histogram are adjusted by imposing the following relation: $\sum_{i=0}^{n_{bins}}{\hat{n}}_{l}=1$. Finally, Table 3 reports the minimum and maximum diameters observed in the PSD by both experiments and NanoDOME PBM calculations. The validation is performed by comparing the predicted distributions with experimental data.

Due to the non-replicable nature of the experimental reactor and the lower values of NDs processed by NanoDOME, a subtle fluctuation of the predictions is expected, especially for the PBM given its stochastic nature. Furthermore, NPs’ time evolution is expected to be primarily influenced by the temperature along the streamline. Especially for the high temperature cases e.g., 2, 3, and 4, high temperature zones where the supersaturation rate is lower than one $\left(S_{s}^{sat}<1\right)$ are possible. This spottily distributed behavior is expected to be observed mainly in the early stages of the NPs’ thermophysical evolution, manifesting increasing oscillations in magnitude as the temperature grows, and leading to partial re-evaporation phenomena. Furthermore, computing the PSD for lower NDs might lead to a widened PSD with respect to the experiment. Despite this, the proposed post-processing can provide detailed information on how the thermophysical transformation of mesoscopic entities takes place.

### 3.1. PSD Streamline-Weighted Mean Value over Time—Primary Particles (PBM and MoM)

#### 3.1.1. Temperature Effects—Cases [1, 2, 3]

The temporal evolution of $\bar{d}$ for cases 1, 2, and 3 is shown in Figure 5. The observed trends clearly suggest a positive correlation between average diameter and temperature. At higher temperatures, particles face a steeper initial increase in their diameter before reaching a stable size, because the growth phenomena are characterized by a faster rate.

The accelerated growth is directly connected to the particles’ temperature history: the higher the starting temperature, the longer the time window in which the particles face a temperature that activates condensation and coagulation phenomena. After the nucleation event, the particles grow quickly until, upon reaching the cold collection surface, they steadily maintain their steric properties. The MoM and PBM models show consistent thermophysical evolution paths. The MoM in Figure 5 (left) shows an initial step-like behaviour which rockets the mean diameter value to around 3.75 nm. This odd peculiarity of the initial peak is merely due to a numeric instability because of the selected time step $\Delta t=10^{-7}\;s$ set for the solution of the GDE (a good compromise between timescale capturing capability and computational effort) and the temporal numerical scheme adopted. Such a trend immediately disappears after the initial time step and does not affect the final results.

#### 3.1.2. Mass Flow Effects—Cases [2, 5]

Figure 6 shows the temporal evolution of $\bar{d}$ for cases 2 and 5. Its dependence on $m_{He}$ clearly reveals an effect on the very early stages of the mesoscopic entity’s thermophysical evolution. As stated previously, the higher the mass flow rate of the carrier gas, the stronger the cooling effect given by convection. Accordingly, the case characterized by a higher flow rate shows a sensibly lower asymptotic value for the particles’ diameters, as is also reported in Table 2. Moreover, we observe an anticipation of primary particle formation for the higher flow rate. This suggests that a stronger cooling anticipates the nucleation events because the supersaturation condition is attained more rapidly. On a qualitative side, despite the potentially smaller NPs obtainable at higher flow rates, the effect of temperature still dominates these processes. Its strong influence on the saturation pressure $p^{sat}\left(T\right)$ directly influences the supersaturation rate $S_{s}^{sat}$.

#### 3.1.3. Pressure Effects—Cases [3, 4]

By increasing the pressure 10-fold at constant He mass flow and temperature (Case 3 vs. Case 4, Figure 7), the number of interactions between NPs is expected to grow significantly. However, both MoM and PBM predict only a moderate increase in the asymptotic $\bar{d}$ value for the high-pressure case. This suggests that, although the number of interactions and potentially new formation processes might increase, the pressure change does not overcome the leading role of temperature in GPNS, both qualitatively and quantitatively.

To summarize, the comparison between the calculations and the experiment reported in Table 2 for all five cases shows the following.

The MoM tends to underestimate the average diameter and the standard deviation, especially for small particles; for instance, a too small value of 1.5 nm is obtained for the lowest temperature, $T_{h}=1260$ K.

The PBM tends to slightly overestimate the average diameter for cases 2, 3, and 4, but the agreement with the experiment is quite good. The values match within one standard deviation. A noticeable diameter underestimation for cases 1 and 5 can be observed. Such behaviour is mainly due to the CFD simulation, in which low temperature or high velocity zones can be overestimated, which, thus, leads to a smaller time window for nanoparticle growth. This is related to the low replicable nature of the reactor, in which convective and transport phenomena can exhibit a random and non-reproducible behaviour by a Reynolds averaged Navier–Stokes (RANS) method which is, currently, the only viable approach with respect to computational time. This effect is strongly reduced for cases 2, 3, and 4, in which the high temperature produces an silver vapour pressure higher than the saturation limit, while the gas flow induces a local Reynolds number in which the convective phenomena show a fairly predictable behaviour.

#### 3.1.4. Final Distributions—Primary Particles (PBM)

To further understand the silver NP synthesis from IGC at a quantitative level, the comparison between NanoDOME’s PBM PSD predictions and the experimental data can clarify the influence of thermodynamic and operative conditions on primary PSD. The computed and experimental PSDs are reported in Figure 8, Figure 9, Figure 10 and Figure 11 along with the best-fit lognormal distributions. Moreover, in Table 3, the lower and upper end values of the PSDs are reported as a further comparison between PBM calculations and experimental data.

The representative cases selected from the PBM’s DOE to compute the PSD are the ones characterized by the maximum value of one of the three controllable parameters of the reactor, i.e., case 3 for temperature, case 4 for pressure, and case 5 for the mass flow rate, alongside the reference case 2.

**Table 3:** Minimum and maximum values that define the upper and lower end of the distribution calculated by PBM or observed experimentally.

	NanoDOME—PBM $d_{min}\;[\mathrm{nm}]$	NanoDOME—PBM $d_{max}\;[\mathrm{nm}]$	$\mathrm{Exp}\;d_{min}\;[\mathrm{nm}]$	$\mathrm{Exp}\;d_{max}\;[\mathrm{nm}]$
Case 2	3.1	25.8	4.5	21.1
Case 3	5.1	47.5	8.1	26.8
Case 4	3.7	64.0	5.1	47.6
Case 5	2.9	19.2	7.1	18.6

The inspection of Figure 8, Figure 9, Figure 10 and Figure 11 and Table 3 reveals that NanoDOME PSDs are generally wider than the experimental ones. The reason is merely stochastic and linked to the number of events processed by NanoDOME, which are a small population compared to the experiment. Nevertheless, the particle size ranges spun by NanoDOME predictions and experimental data are quite similar. The PSDs shift to higher values with increasing temperature or He pressure, whereas by increasing the He mass flow rate, a shift toward lower values is observed, as expected.

The pressure increase effect, comparing case 4 to case 3, clearly suggests a pressure-induced widening of the range $d_{max}-d_{min}$, with $d_{min}$ almost unaffected by pressure. This effect can be justified by the increase in collisional events inside the gas phase, with a resulting larger probability for the mesoscopic entities to undergo further growth processes and reach larger diameters; indeed, for case 4, the PBM predicted $d_{max}$ reaches 60 nm, leading to a flatter PSD and, therefore, to an overestimation of the pressure effect. Despite this, the denser region of the PSD exhibits a similar range, 10<d<30 nm, for the two pressures.

The He mass flow rate effect suggests an anticipated NP formation for case 5 (higher flow) compared to case 2 (lower flow), due to the GP cooling effect discussed previously. The quantitative consequences on the final NPs’ size distribution are a narrower range of values and a shift toward smaller diameters, as can be verified by looking at Table 3. The higher the flow, the quicker the NPs attain a size plateau where they stop growing. The dominant influence of temperature on the process, as highlighted by the previous set of results, is confirmed. In fact, the predicted PSDs for case 2 and 5 lie in the same range, with only a slight shift in the latter toward a lower diameter in spite of the doubled flow rate.

## 4. Discussion

In this work, the influence of thermodynamic conditions on primary particles’ thermophysical evolution and final diameter distribution was analyzed for a GPNS reactor. The experimental data were compared with the predictions of NanoDOME’s computations. The main goal was to characterize both qualitatively and quantitatively the role of temperature, pressure, and He mass flow rate on primary particles’ evolution and final size along the computational domain. The results obtained from the experimental reactor, despite its intrinsically non-replicable flow patterns, provided a solid validation approach. NanoDOME proved to be a reliable and consistent toolkit, whose predictions show general good agreement with the experimental data, although the pressure increase effect prediction must be improved. Regarding the mesoscopic models adopted in this study, the PBM tool turned out to be more accurate than MoM. The stochastic approach exploited by the former proved to be a very powerful way to tackle the semi-randomness of the experimental reactor flow patterns. Moreover, the MoM showed a general underestimation of the average diameter. Both MoM and PBM qualitatively agree concerning the evolution of the average size along the computational domain, demonstrating phenomenological and physical consistency.

Given the good matching between NanoDOME’s predictions and experimental data, particularly for PBM, a further step toward the improvement of the framework could be adopting an iterative coupling algorithm for MoM to increment its accuracy. Furthermore, a third modelling approach of the mesoscopic scale, based on a coarse-grained molecular dynamic (CGMD), could be linked to the continuum reactor scale. Such a model could provide further insights into the aggregates’ internal structures by means of fractal dimensions and graphical representation.

## Figures and Tables

**Table 2:** Final value of $\bar{d}$ [nm] and σ for the experimental and NanoDOME calculations.

Mean Diameter [nm] and Standard Deviation	Case 1 $\left(1260\;K,\;260\;\mathrm{Pa},\;1.8\times 10^{-7}\;\mathrm{kg}/s\right)$		Case 2 $\left(1403\;K,\;260\;\mathrm{Pa},\;1.8\times 10^{-7}\;\mathrm{kg}/s\right)$		Case 3 $\left(1466\;K,\;260\;\mathrm{Pa},\;1.8\times 10^{-7}\;\mathrm{kg}/s\right)$		Case 4 $\left(1466\;K,\;2600\;\mathrm{Pa},\;1.8\times 10^{-7}\;\mathrm{kg}/s\right)$		Case 5 (1403 K, 260 Pa, 3.6$\times 10^{-7}$ kg/s)	
	$\bar{d}$	σ	$\bar{d}$	σ	$\bar{d}$	σ	$\bar{d}$	σ	$\bar{d}$	σ
Experimental	5.70	2.31	10.7	3.66	14.8	3.43	22.1	4.46	10.8	2.30
MoM	1.50	0.79	7.90	0.79	15.8	0.74	18.1	0.74	4.17	0.80
PBM	3.64	1.57	11.4	4.80	18.2	4.01	21.2	3.99	8.02	3.03

## Data Availability

Data will be available upon reasonable request.

## References

[1] Malekzadeh M., Malekzadeh S., Mark T. (2021). Vapor-phase production of nanomaterials. Chem. Soc. Rev..

[2] Granqvist C.G., Buhrman R.A. (1976). Ultrafine metal particles. J. Appl. Phys..

[3] Gleiter H. (2000). Nanostructured materials: Basic concepts and microstructure. Acta Mater..

[4] Raffi M., Rumaiz A.K., Hasan M.M., Shah S.I. (2007). Studies of the growth parameters for silver nanoparticle synthesis by inert gas condensation. J. Mater. Res..

[5] Hongyu L., Jun L., Daohua S., Tareque O.W., Jiale H., Qingbiao L. (2014). Modeling of Silver Nanoparticle Formation in a Microreactor: Reaction Kinetics Coupled with Population Balance Model and Fluid Dynamics. Ind. Eng. Chem. Res..

[6] Galleni F., Strappaveccia F., Ghedini E. (2019). A novel approach for the estimation of nanoparticle evaporation through the Method of Moments. J. Phys. Conf. Ser..

[7] OpenCFD. OpenFOAM. ESI Group. https://www.openfoam.com/.

[8] Yun X., Pratsinis S.E. (1991). Gas phase production of particles in reactive turbulent flows. J. Aerosol Sci..

[9] Alma Mater Studiorum University of Bologna T3.1-NanoDOME2020-NMP-2014-Two-Stage. https://cordis.europa.eu/project/id/646121/results/it.

[10] Strappaveccia F., Galleni F., Ghedini E. (2019). 2020 NanoDome Project: A Unified Approach for Gas-Phase Nanoparticles Synthesis Modelling. J. Phys. Conf. Ser..

[11] Bandyopadhyaya R., Lall A.A., Friedlander S.K. (2004). Aerosol dynamics and the synthesis of fine solid particles. Powder Technol..

[12] Wang C.-S., Friedlander S., Mädler L. (2005). Nanoparticle aerosol science and technology: An overview. China Particuology.

[13] Eggersdorfer M.L., Pratsinis S.E. (2014). Agglomerates and aggregates of nanoparticles made in the gas phase. Adv. Powder Technol..

[14] Sanibondi P. (2015). Numerical investigation of the effects of iron oxidation reactions on the fume formation mechanism in arc welding. J. Phys. D Appl. Phys..

[15] Friedlander S.K. (2009). Smoke, Dust and Haze.

[16] Menter F.R., Kuntz M., Langtry R. (2003). Ten years of industrial experience with the SST turbulence model. Fourth International Symposium on Turbulence, Heat and Mass Transfer.

[17] OpenCFD. https://www.openfoam.com/documentation/guides/latestapiclassFoam_1_1sutherlandTransport.html.

[18] Pasquini L., Barla A., Chumakov A.I., Leupold O., Rüffer R. (2002). Size and oxidation effects on the vibrational properties of nanocrystalline α-Fe. Phys. Rev. B-Condens. Matter Mater. Phys..

[19] Signorini L., Pasquini L., Savini L., Carboni R., Boscherini F., Bonetti E., Giglia A., Pedio M., Mahne N., Nannarone S. (2003). Size-dependent oxidation in iron/iron oxide core-shell nanoparticles. Phys. Rev. B-Condens. Matter Mater. Phys..

[20] Patelli N., Migliori A., Morandi V., Pasquini L. (2020). Interfaces within biphasic nanoparticles give a boost to magnesium-based hydrogen storage. Nano Energy.

[21] Rossi G., Calizzi M., Di Cintio V., Mahkos S., Admidani L., Pasquini L., Boscherini F. (2016). Local Structure of V Dopants in TiO_2_ Nanoparticles: X-ray Absorption Spectroscopy, Including Ab-Initio and Full Potential Simulations. J. Phys. Chem. C.

